# NKG2A Down-Regulation by Dasatinib Enhances Natural Killer Cytotoxicity and Accelerates Effective Treatment Responses in Patients With Chronic Myeloid Leukemia

**DOI:** 10.3389/fimmu.2018.03152

**Published:** 2019-01-17

**Authors:** Ming-Chin Chang, Hung-I Cheng, Kate Hsu, Yen-Ning Hsu, Chen-Wei Kao, Yi-Fang Chang, Ken-Hong Lim, Caleb Gonshen Chen

**Affiliations:** ^1^Department of Hematology, MacKay Memorial Hospital, Taipei, Taiwan; ^2^Department of Medicine, MacKay Medical College, New Taipei, Taiwan; ^3^Department of Hematology, MacKay Memorial Hospital, Hsin-Chu, Taiwan; ^4^Department of Medical Research, Transfusion Medicine & Immunogenetics Laboratories, MacKay Memorial Hospital, Tamsui, Taiwan; ^5^GCRC Laboratory, Department of Hematology, MacKay Memorial Hospital, New Taipei, Taiwan

**Keywords:** CML, BCR-ABL, major molecular response (MMR), deep molecular response (DMR), natural killer cell, NKG2A, p38 mitogen-activated protein kinase

## Abstract

Chronic myeloid leukemia (CML) is a hematological malignancy characterized by the presence of *t*(9;22) chromosomal translocation that results in *BCR-ABL* fusion gene. ABL tyrosine kinase inhibitors (TKIs), such as imatinib, nilotinib, and dasatinib, are currently the front-line treatment options for CML. Recently, natural killer (NK) cell activation and expansion have been shown to be associated with optimal treatment responses for CML. To investigate the effects and mechanisms of these TKIs on NK cells, here we characterized activating and inhibitory NK receptors in CD3^−^CD16^+^CD56^dim^ NK cells isolated from CML patients in chronic phase (CP). The expressions of activating NK receptors, such as NKG2D, natural cytotoxicity receptor (NCR) and DNAM-1, rebounded after successful TKI treatments for CML. In contrast, among the three surveyed inhibitory receptors (NKG2A, KIR2DL1, and KIR3DL1), only the expression of NKG2A was reverted and suppressed to a very low level by dasatinib, and not by imatinib or nilotinib. CML patients treated with dasatinib indeed expressed fewer NKG2A+ NK cells, which send negative signals for induction of NK cytotoxicity. For these dasatinib-treated patients, the duration to reach major molecular response (MMR) was shorter, and significantly correlated with individual's NKG2A+ NK cell number. This clinical relevance to NKG2A was not observed in treatments with imatinib or nilotinib. In line with dasatinib-specific down-regulation of NKG2A, NK cytotoxicity evaluated by the killing assay was also significantly higher in patients treated with dasatinib than in those treated with imatinib or nilotinib. The lower NK cytotoxicity from imatinib or nilotinib treatments could be reverted by NKG2A blockade using anti-NKG2A antibody. Further *in vitro* experiments revealed mechanistically that dasatinib could inactivate p38 mitogen-activated protein kinase (MAPK), and consequently affect nuclear import of GATA-3 and GATA-3 transcriptional activities for NKG2A. Our results highlight the dual effects of dasatinib in direct inhibition of ABL kinase and in immunomodulation through NKG2A down-regulation, contributing to accelerated molecular responses (MR) in CML.

## Introduction

CML is a myeloproliferative disorder of hematopoietic stem cells harboring *t*(9;22) chromosomal translocation known as Philadelphia chromosome (Ph) (1). This translocation introduces a break point cluster (*BCR*) on chromosome 22 that fuses with Abelson murine leukemia viral oncogene homolog 1 (*ABL*) tyrosine kinase of chromosome 9 (2, 3). Over the past two decades, anti-CML treatments with tyrosine kinase inhibitors (TKIs), which include imatinib, nilotinib, and dasatinib, have drastically improved overall patient survival (4–6). Based on multivariate analyses, failures in treatment adherence and in achieving major molecular responses (MMR) are the only two independent predictors for loss of complete cytogenetic response (CCyR) in TKI treatments for CML (7). MMR and CCyR can be considered as surrogate outcomes for overall survival in the first-line treatments for CML (8). Intriguingly, CML patients who had an expansion of mature NK cells after TKI treatments could successfully discontinue the treatments, as suggested in recent studies, emphasizing the important role of immunosurveillance by mature NK populations (9, 10).

An interesting observation during dasatinib treatments for CML is that patients who develop expansion of large granular lymphocytes (LGLs) reached MMR or deep molecular responses (DMR) faster than those who did not have expansion of LGLs (11). LGLs consist of mono- or oligo-clonal cytotoxic T cells and NK cells (12). Indeed, various strings of evidence have linked LGL expression to the molecular responses to the treatment with dasatinib, but not with imatinib or nilotinib (11–13). Further, several groups reported that dasatinib markedly enhances cytokine production and cytotoxic function of NK cells in CML patients (14, 15). For comparison, the increase of CD3^−^CD16^+^CD56^+^and mature CD56^+^CD57^+^cells could not be achieved with imatinib or nilotinib (16). Moreover, Hughes and colleagues reported that when TKI-treated, CML patients reached MMR or molecular response^4.5^ (MR^4.5^), their NK cell profiles displayed a larger proportion of mature, cytolytic CD57^+^CD62L^−^NK cells, with repertoires of activating and inhibitory receptors similar to that found in healthy people (17). However, the study by Hughes et al. did not differentiate expressions of NK receptors affected by the different TKI treatments for CML.

Major NK activating receptors that function in cancer cell recognition include C-type lectin receptor NKG2D, the Natural Cytotoxicity Receptor (NCR) family (NKp30, NKp46, and NKp44), and the co-activating receptor/ adhesion molecule DNAM-1 (18). These activating receptors are constitutively expressed on both mature NK subsets: CD56^dim^ cells with cytolyic activities and CD56^bright^ (CD56^br^) cells with immunoregulatory activities (19). High surface expressions of NKG2D and DNAM-1 ligands are found on leukemia blasts harboring *BCR-ABL*, making these blasts more susceptible to NK-dependent lysis than the blasts lacking these ligands (20). But the treatments with imatinib, nilotinib, or dasatinib, also result in decreased expression of NKG2D ligands, which include MHC-related Ags-A/B (MICA/B) and UL-16 binding proteins; this in turn limits NK cell-mediated lysis (21, 22).

Alternatively, NK cells can be activated by suppression of inhibitory signaling. Malignant cells that lose HLA class I (HLA-I) molecules may be recognized by inhibitory killer cell immunoglobulin-like receptors (KIRs) that are specific for HLA-A, B, C, and F molecules (23, 24), and by CD94/NKG2A that is specific for HLA-E (25, 26). Since HLA-E is expressed by most normal and neoplastic hematopoietic cells, its presence protects most cells from CD94/NKG2A+ NK-mediated killing (27–29). In the pharmaceutical development of Lirilumab and Monalizumab, these monoclonal antibodies block the KIR/HLA-C and NKG2A/HLA-E, respectively, and have been shown to enhance specific NK-mediated killing against HLA-I deficient tumor cell lines in preclinical models (30, 31).

Although many reports suggested a crucial role of NK activities in TKI treatment responses for CML, how these BCR-ABL inhibitors modulated expressions of NK receptors had not been characterized. This study began the quest, and identified a new anti-cancer mechanism of dasatinib that was through down-regulation of inhibitory receptor NKG2A. Reduction of NKG2A+ NK cells by dasatinib, not by imatinib or nilotinib, enhanced overall NK cytotoxicity, which was significantly associated with the effectiveness of treatment responses in CML. We further dissected the molecular processes that involved dasatinib-specific inhibition of p38 mitogen-activated protein kinase (MAPK), and subsequent inhibition of GATA-3 on its phosphorylation, nuclear import, and transcriptional activities. Phosphorylation of GATA-3 on serine residue 308 by p38 MAPK is required for GATA-3 to be imported into the cell nuclei (32). GATA-3 is a transcription factor important in NK cell differentiation and function, specifically in the processes of NK cell maturation, NK homing to the liver, and IFN-γ production (33). Importantly, GATA-3 binds to the promoter of *NKG2A* to facilitate *NKG2A* gene expression (34), which however could be inhibited indirectly by dasatinib, as revealed in this study. Therefore, in addition to BCR-ABL inhibition, dasatinib also affected NKG2A expression to promote NK cytotoxicity against CML.

## Materials and Methods

### Patients, Controls, and Samples

This study recruited 88 Ph+ CML-CP patients under standard treatment regimen with imatinib (*n* = 21), nilotinib (*n* = 37), or dasatinib (*n* = 30) (Table 1: patient demographics). For each patient, the median average daily dose per day was 100 mg dasatinib (ranged 10–140 mg), 400 mg imatinib (ranged 200–400 mg), or 600 mg nilotinib (ranged 75–800 mg). During the follow-up period, no patients switched or discontinued TKIs, but there might be modification of the dose due to side effects of the TKIs. Twenty-one age-matched healthy adults (HA) were studied in parallel as the controls. Peripheral blood (PB) samples of patients were collected multiple times for quantification of *BCR-ABL* transcripts, as previously described (35). MMR is defined as ≥3 log reduction of the BCR-ABL product on the international scale, and deep molecular response (DMR) is MR^4.0^ at≥4 log reduction. Pre-MMR values are *BCR-ABL* transcript levels >0.1% or <10%. Bone marrow (BM) core biopsy and aspiration were performed for cytogenetic study. Mononuclear cells (MCs) from PB or BM were isolated by Ficoll-Paque Plus (Amersham, UK) gradient centrifugation and cryopreserved until use. Sampling for NK cells analyses from CML patients at initial diagnosis was prior to TKIs therapy and was done after taking daily TKIs in the morning. This study was approved by the Mackay Memorial Hospital Institutional Review Board (18MMHIS113), and was carried out in accordance with the principles of the Declaration of Helsinki.

**Table 1:** Demographics of the recruited patients with CML in chronic phase.

	**Healthy adults (*n* = 21)**	**Imatinib (*n* = 21)**	**Nilotinib (*n* = 37)**	**Dasatinib (*n* = 30)**	**Imatinib/Nilotinib*p*-value**	**Dasatinib/Imatinib*p*-value**	**Dasatinib /Nilotinib*p*-value**
**Age, median (range), years**	49 (29–62)	51 (28–84)	50 (29–80)	50 (23–80)	NS	NS	NS
**Sex, %**							
Male	30	52	51	57			
Female	70	48	49	43			
**Receiving Dose (*****n*****; %)**							
	NA	≥400 mg (15; 71%)	≥600 mg (25; 69%)	≥100 mg (20; 67%)			
	NA	<400 mg (6; 29%)	<600 mg (11; 31%)	<100 mg (10; 33%)			
**TKI duration to MMR, [median(range), months]**							
MMR (≥3 log reduction; ≤ 0.1^IS^)	NA	31 (7–128)	17 (4–90)	10 (3–94)	NS	<0.0001	0.037
DMR (≥4 log reduction; ≤0.01^IS^)	NA	37 (18–128)	22 (11–115)	20 (9–61)	NS	0.006	NS
**TKI therapy, %**							
Pre-MMR	NA	10	19	30			
MMR (≥3 log reduction; ≤0.1^IS^)	NA	90	81	70			
DMR (≥4 log reduction; ≤0.01^IS^)	NA	76	51	50			

### Flow Cytometry

To quantify various NK cell populations in the PBMCs, 5 × 10^5^ cells were stained with various combinations of fluorophore-conjugated monoclonal antibodies (mAbs). The Gating process of CD3^−^CD16^br^CD56^dim^NK cells was shown in Figure S1, according to the previous report (36). Stained cells were fixed with 4% paraformaldehyde and examined by FACSCalibur (BD Biosciences), and the data analyzed Cell Quest Pro software (FlowJo, LLC, Ashland, OR). A total of 50,000 lymphoid events were acquired in each sample. Cells were phenotypically analyzed by staining for 20 min with the following fluorophore-conjugated anti-human mAbs: fluorescein isothiocyanate (FITC)-αCD3, PerCP eFluor710-αCD16, phycoerythrin (PE)-CD56, eFlour660-CD107a, APC-CD158 (KIR2D L1/S1/S3/S5), APC-CD158e1 (KIR3DL1), APC-CD159a (NKG2A), APC-CD226 (DNAM-1), APC-CD244 (2B4), APC-CD314(NKG2D), AF647-CD337 (NKp30), and APC-anti-HLA-E (all from BioLegend, eBioscience, and Miltenyi Biotec). Positive staining populations were determined by comparison with isotype controls. To evaluate HLA expression on CD45^+^CD34^+^ myeloid progenitor cells, the cells were stained with PerCP Cy5.5-CD45 (BioLegends), PE-CD34 mAbs (BD Biosciences), and APC-anti-HLA-E mAb, and examined by flow cytometry.

### Cell Culture

K562 cell line was purchased from ATCC (CCL-243). Primary NK cells were isolated using magnetic bead approaches according to the manufacturer's protocol (NK kit 11349D, Invitrogen). K562 cells and primary NK cells were cultured in RPMI-1640 media (Life Technologies) containing 10% fetal bovine serum (FBS) and 1% penicillin-streptomycin-amphotericin B (Life Technologies). NK92 cells (BRC, HC2003) were cultured in αMEM media (Gibco) supplemented with 12.5% FBS, 12.5%horse serum, 0.2 mM Myo-inositol (Sigma-Aldrich), 0.1 mM 2-mercaptoethanol (Sigma-Aldrich), 0.02 mM folic acid (Sigma-Aldrich), and 200 U/mL recombinant human IL-2 (PeproTech). To analyze the effects of the TKIs on NK cell functions, imatinib (2 μM), nilotinib (2 μM), and dasatinib (100 nM) purchased from Cayman Chemicals were tested *in vitro*. To inhibit p38 MAPK activities, SB203580 (1 μM; Cayman Chemical) was used.

### Cell Transfection

To make stable cell lines with HLA-E expression, lentiviral vectors (pLenti-GIII-CMV-RFP-2A-Puro) containing human *HLA-E* were purchased from Applied Biological Materials (Heidelberg, Germany). K562 cells were transduced with the lentiviral vectors to express HLA-E, and named K562-ecells. For NKG2A knock-down, pLKO.01-puro KLRC1lentiviral vectors harboring small hairpin RNA (shRNA)-targeting *NKG2A*were purchased from Academia Sinica (Taipei, Taiwan). The cells were transfected for 48 h, and then the medium was replaced with fresh culture medium containing 300 μg/ml puromycin (Sigma-Aldrich, MO), which allows for cell clonal selection. Cells with transient *P38* knockdown (KD) was prepared using lentiviral vectors that contain p38-targeting small interfering RNA (siRNA) (Cell Signaling Technology, Danvers, MA). To confirm the effects of p38 knockdown, mouse anti-p38 (Merck Millipore, Germany) was used to evaluate P38 expression levels.

### Immunoblotting

Cells were lysed in RIPA buffer, and whole-cell extracts were quantified by the Bradford assay (Bio-Rad). For assessment of nuclear proteins, nuclear extracts were obtained using NE-PER nuclear and cytoplasmic extraction kit (Thermo Scientific). The protein samples or cell lysates were analyzed by SDS-PAGE and Western blot. Briefly, after proteins were transferred onto PVDF membranes (Millipore), the membranes were incubated with indicated primary antibodies, followed by a HRP-conjugated secondary antibody. Immunoreactive bands were detected using the Western Lighting Plus-ECL system (PerkinElmer) or the SuperSignal West Dura Extended Duration Substrate (Pierce). The primary antibodies used for Western blot included anti-p38 (2F11, Millipore), anti-phospho p38 (Thr180/Tyr182) (2BB10, Cell Signaling), and anti-β-actin (C4, Millipore), anti-NKG2A (Abnova), anti-GATA-3 (D13C9, Cell Signaling), anti-phospho-GATA-3 (Ser308) (EPR18118, Abcam), and anti-Histone H3 (BioLegend).

### Killing Assay

The cytotoxicity assay was performed by flow cytometry as previously described (37), with slight modification. K562-e target cells were labeled with carboxyfluorescein succinimidyl ester (CFSE) at a final concentration of 2 μM (Abcam); this discriminated target cells from effector cells. NK cells were incubated with CFSE-labeled K562-e target cells at different effector-to-target (E:T) ratios ranging from 25: 1, 12: 1, to 6: 1, in 96-well plates. The cells were cultured in 150 μL culture media, and 50,000 target cells were used constantly. The positive control—NK92 cell line (effector), was tested with K562-ecells (target) at an E: T ratio of 25: 1. The negative-controls were K562-e cells alone, and were included in each test. After 4-h co-culture at 37°C, 5% CO_2_, the cell mixture was stained with 5 μL of 7-AAD (BD Biosciences) for 15 min in the dark. For the blocking experiments, the target cells were treated withanti-CD32 (AF1330-SP, R&D Systems) and anti-CD64 (MAB1257-SP, R&D Systems) at 10 mg/mL each for 30 min at 4°C. Flow cytometry data were analyzed on FACSCalibur (BD bioscience). NK cytotoxicity (%) was calculated as the percentage of the cells positive for both CFSE and 7-AAD in total CFSE positive cells, excluded % spontaneous lysis that was estimated from the negative controls (Figure S2).

### Statistical Analysis

To compare the means between two independent groups that were not normally distributed, the non-parametric Mann-Whitney U test was used. If two groups were normally distributed, Student's *t*-tests were applied to test for comparison. To compare three or more independent groups, one-way analysis of variance (ANOVA) with Bonferroni's multiple comparisons were used. The paired *t*-test was used to help assess the effect of a TKI on expressions of NK receptors by comparing patients before (at diagnosis) and after the TKI treatment. The paired *t*-tests was also used to compare NKG2A activities in human NK cells by the treatment with anti-NKG2A neutralizing mAb.^*^P < 0.05 was deemed significant. Cumulative response rates were calculated using the cumulative incidence approach and Mentle-Cox method. For these longitudinal analyses, quantitative variables were dichotomized according to their median value. The cutoff points for the most promising variables were optimized by receiver operating characteristics (ROC) curves and the Youden index. All data of CML patients were typically presented as median ± standard deviation (SD). Experimental data were presented as a pool of three experiments (mean ± standard error). The threshold for statistical significance was defined at *p* < 0.05. GraphPad Prism 6 (GraphPad Software) or SPSS 12.0 (SPSS Inc., Chicago, IL, USA) was used for all the analyses.

## Results

### NK Cell Phenotypes Affected by Different TKI Treatments for CML

To evaluate how NK cell activities in CML-CP patients were affected by different TKIs, the blood samples from 88 recruited patients were collected and analyzed. Table 1 summarizes the clinical characteristics of these patients. Patients included in this study did not change to other types of TKIs or discontinued their TKI treatments. Because of individual variability in tolerance toward TKI side effects, among all recruited patients, 29% of imatinib-treated patients received lower-than-standard doses (400 mg imatinib daily as the standard dose), 31% of nilotinib-treated patients received lower doses (600 mg nilotinib daily as the standard dose), and 33% of dasatinib-treated patients received lower doses (100 mg dasatinib daily as the standard dose).

**Figure 1:**
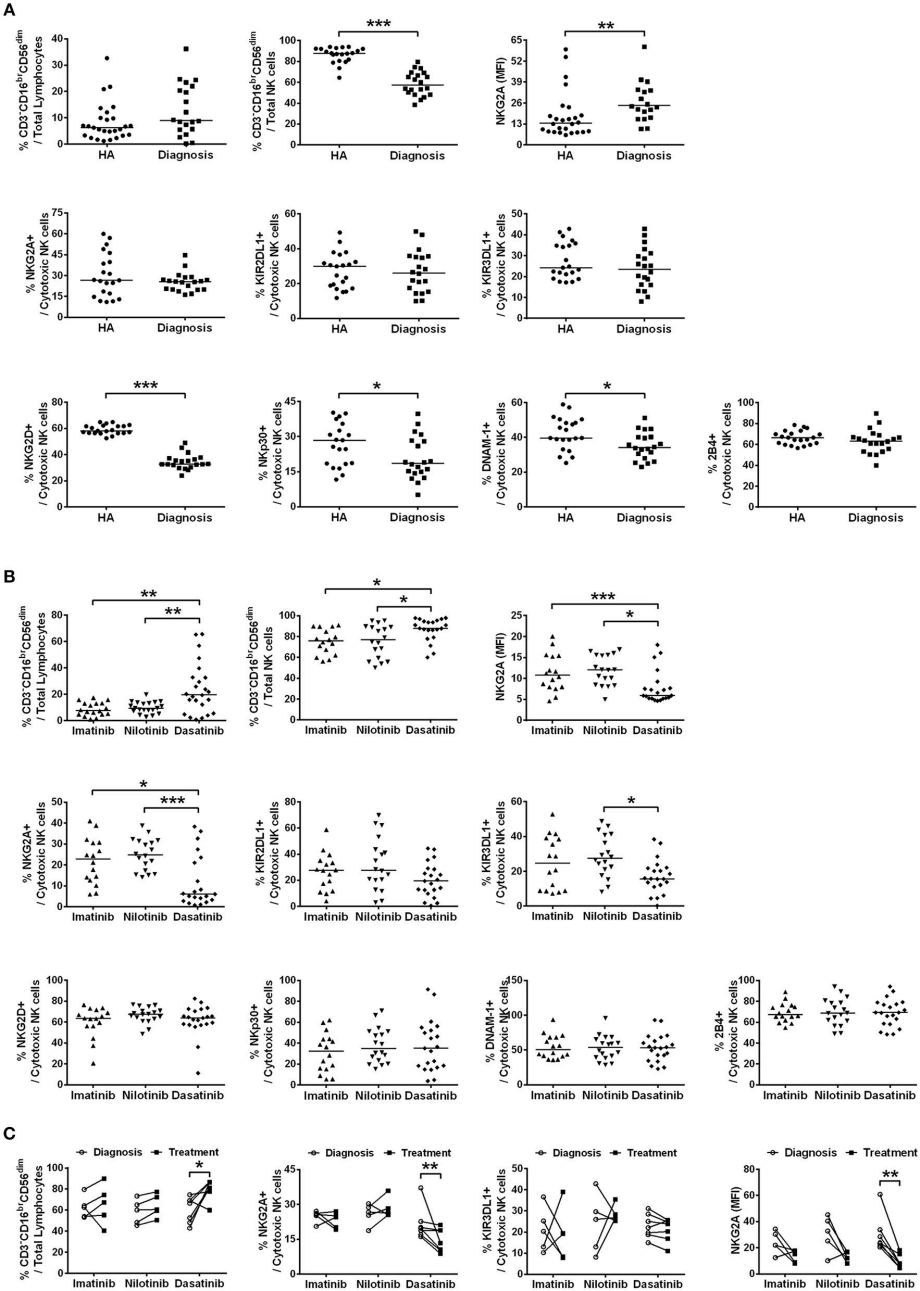
The different TKI treatments for CML resulted in differential expressions of major NK receptors in CD3^−^CD16^br^CD56^dim^ NK cells. **(A)** CML patients at their initial diagnosis had fewer NK cells expressing the activating receptors, compared to age-matched healthy controls (HA). The percentages of cytotoxic NK cells positive for activating receptors (NKG3D, NKp30, DNAM-1, and 2B4) and for inhibitory receptors (NKG2A, KIR2DL1, and KIR3DL1) in total lymphocytes were shown. The levels of NKG2A in NKG2A+ NK cells were presented as mean fluorescence intensity (MFI). The NK cells expressing these inhibitory receptors (NKG2A, KIR3DL1, and KIR3DL1) were not different in cell frequency between healthy people and CML patients at their initial diagnosis. However, the surface density of the inhibitory receptor NKG2A on the NKG2A+ NK cells was significantly higher in CML patients at their initial diagnosis than in healthy controls. Data comparison was performed by the Student's *t*-test; if data distribution was not normally distributed, non-parametric Mann-Whitney *U*-test was used. **(B)** The percentages of cytotoxic NK cells expressing these activating and inhibitory NK receptors in total lymphocytes from different TKI-treated patients when they reached MMR were compared. Data were analyzed using one-way ANOVA with Bonferroni's multiple comparisons. Different TKI treatments did not differentially affect expressions of NK cells with activating receptors, but dasatinib uniquely down-regulated the percentages of NK cells expressing inhibitor receptors—NKG2A and KIR3DL1. Dasatinib also significantly reduced the surface density of NKG2A in NKG2A+ NK cells in these patients. **(C)** Down-regulation of NKG2A-expressing cells and KIR3DL1-expressing cells were further examined by the paired *t-*test in the same patients at two time points: (i) at the initial diagnosis of CML before starting a TKI treatment; (ii) during the TKI treatment when the patients reached MMR. Dasatinib for CML uniquely suppressed % NKG2A+ NK cells, as well as the surface density of NKG2A in NKG2A+ NK cells. Dasatinib also increased % cytotoxic NK cells, whereas imatinib or nilotinib did not affect the population sizes of cytotoxic NK cells. Bars denote the median. Statistically significance was defined by ^*^p < 0.05, ^**^p < 0.01, and ^***^p < 0.001.

We determined the fractions of CD3^−^CD16^br^CD56^dim^ cells in total lymphocytes isolated from patients when they were first diagnosed with CML (the first plots in Figures 1A,B). As the process of immune restoration is dynamic in CML patients at different stages (17), we also performed NK phenotyping when the patients reached MMR. There were no differences in the expressions of CD3^−^CD16^br^CD56^dim^ NK cells in total lymphocytes between healthy controls and newly diagnosed patients with CML, while remarkably lower fraction of this subset in NK-cell populations was observed in CML patients compared to healthy controls (Figure 1A). The treatment by dasatinib significantly enhanced expression of CD3^−^CD16^br^CD56^dim^ NK cells, compared to the treatments by nilotinib or imatinib (the first plot in Figure 1B). Not only was the fraction of CD3^−^CD16^br^CD56^dim^ NK cells in the dasatinib-treated group higher than that in the other TKI-treated groups (Figure 1B), the absolute number of CD3^−^CD16^br^CD56^dim^ NK cells in dasatinib-treated patients was also significantly higher than that in the other TKI-treated groups or the healthy-control group (Figure S3). Due to high myeloid cells in PB of newly diagnosed CML patients, it was conceivable that the absolute number of CD3^−^CD16^br^CD56^dim^ NK cells was significantly lower compared to healthy controls (Figure S3).

We next compared the expression levels of 4 major activating receptors (NKG2D, NKp30, DNAM-1, and 2B4) and 3 major inhibitory receptors (NKG2A, KIR2DL1, and KIR3DL1) on CD3^−^CD16^br^CD56^dim^ cells in newly diagnosed CML patients vs. healthy controls, and in successfully-treated patients who have reached MMR by imatinib, nilotinib, or dasatinib. The percentages of CD3^−^CD16^br^CD56^dim^ NK cells expressing activating receptors NKG2D, NKp30, and DNAM-1 (but not 2B4), all reduced substantially in newly diagnosed CML patients (Figure 1A). But TKI treatments for CML generally reverted or increased expressions of activating NK receptors (Figure 1B; Figure S4). The expressions of activating receptors NKG2D and NKp30 of NK cells were significantly enhanced by TKI treatments. On the other hand, the level of DNAM-1 was promoted only by the second-generation TKIs—nilotinib and dasatinib, and not by the first-generation imatinib. None of the TKI treatments increased the expression of 2B4 (Figure 1B; Figure S4).

In contrast, the percentages of the CD3^−^CD16^br^CD56^dim^ NK cells expressing inhibiting receptors NKG2A, KIR2DL1, and KIR3DL1 were not different between newly diagnosed CML patients and healthy controls (Figure 1A). But among the successfully treated patients who reached MMR, the dasatinib-treated group, not the imatinib-treated or nilotinib-treated group, showed significant reduction in the NK cell populations that expressed inhibitory receptors like NKG2A and KIR3DL1 (Figure 1B).

We thus used the paired *t*-test to further evaluate the impacts of individual TKIs during the treatments for CML. Figure 1C compared NK expression levels when patients were first diagnosed with CML and when they reached MMR. The treatment by dasatinib uniquely enlarged the population of CD3^−^CD16^br^CD56^dim^ NK cells, and at the same time, reduced the fraction of NKG2A+ cells in CD3^−^CD16^br^CD56^dim^ cells and the protein levels of NKG2A on the NKG2A+ cell surface (Figure 1C). For comparison, TKI treatments by imatinib or nilotinib neither enhanced the size of CD3^−^CD16^br^CD56^dim^ NK cell population, nor promoted the expression of NKG2A (Figure 1C).

By detailed phenotyping of major NK receptors in NK cells isolated from treated CML patients, we confirmed previous findings and also observed that global NK activities were reduced at the onset of CML, along with reduction of major NK activating receptors like NKG2D, NKP30, and DNAM-1, and with increase of inhibitory receptor NKG2A levels (Figure 1A). Intriguingly, dasatinib suppressed NKG2A most in the treated CML patients (Figure 1C). In contrast, the other two inhibitory receptors KIR2DL1 and KIR3DL1 surveyed were neither affected by the onset of CML nor by any of the TKIs (Figure 1). These results suggested that enhanced cytotoxic NK cell expression in dasatinib-treated CML patients could be due to the unique down-regulation of NKG2A by dasatinib.

### The Efficacies of TKI Treatments Associated With NKG2A+CD3-CD16^br^CD56^dim^ NK Cells

**Figure 2:**
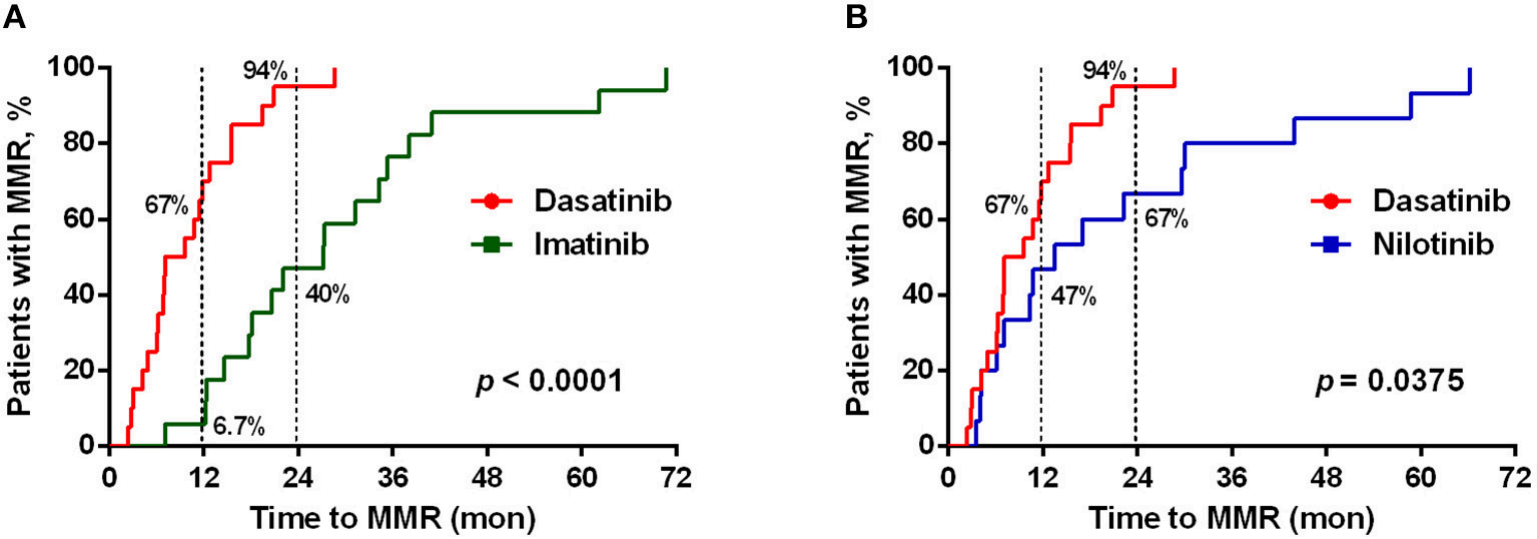
Different TKI treatments resulted in differential cumulative rates of reaching the major molecular responses (MMR). **(A)** The treatment efficacies (in terms of the time to reach MMR) by dasatinib (*n* = 21) and by imatinib (*n* = 19) were compared. **(B)** Comparison between the treatments by dasatinib (*n* = 21) and by nilotinib (*n* = 30). The data were compared at the two time points (12 and 24 months of the TKI treatments), and *P*-values were calculated using the cumulative incidence approach and the Mentle-Cox method.

As previously reported (11, 12, 38), dasatinib exhibits a unique ability to induce LGL expansion, which is correlated with better prognosis and favorable MR in patients with CML. We thus analyzed the differences in patients reaching MMR cumulatively between dasatinib-treated vs. imatinib-treated groups (Figure 2A: *p* < 0.0001), and between dasatinib-treated vs. nilotinib-treated groups (Figure 2B: *p* = 0.037). The percentages of the patients who reached MMR within 12 months were 6.7% (imatinib), 47% (nilotinib), and 67% (dasatinib). The proportions of the patients who reached MMR within 24 months were 40% (imatinib), 67% (nilotinib), and 94% (dasatinib). The relatively lower percentage of imatinib-treated patients reaching MMR might be related to its lower potency of ABL inhibition, when compared with nilotinib and dasatinib (37, 39). In our cohort, one-third of the patients could not tolerate the standard doses of TKIs used in the IRIS (4), ENEST (6), or DASISION (5) trials. Although ~30% of the patients treated with suboptimal doses of TKIs, most MMR was successfully achieved by the dasatinib treatments. DMR was defined as less than MR^4.0^, but not MR^4.5^ or deeper, in this study. Patients treated with dasatinib or nilotinib reached DMR faster (50–51 months in median) than patients treated with imatinib did (76 months in median) (Table 1). This study also confirmed the high efficacy of BCR-ABL inhibitors for treating Taiwanese CML patients. Over 50% of the TKI-treated patients reached DMR by the end of this study.

**Figure 3:**
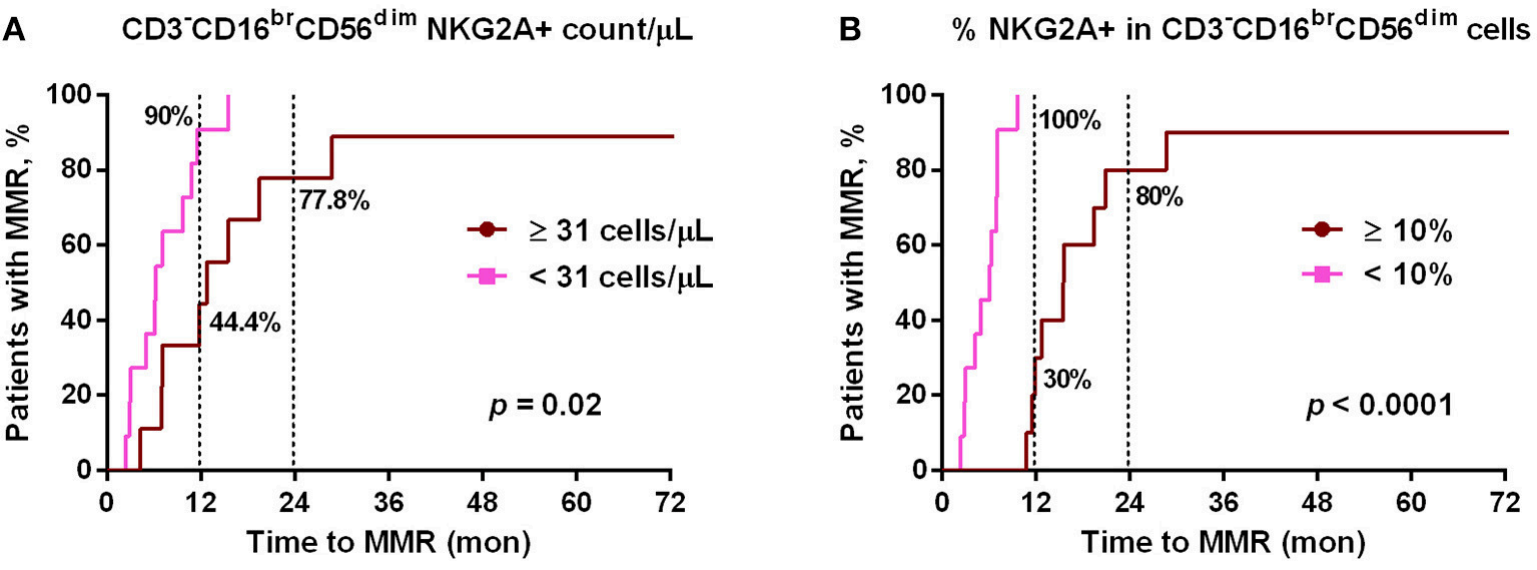
The cumulative rates to reach major molecular responses (MMR) were correlated with dasatinib-mediated down-regulation of NKG2A+ cytotoxic NK cell expression. **(A)** Dasatinib-treated patients were divided into two groups based on the number of NKG2A+ cytotoxic NK cells per μL (31/μL as the optimal cutoff, according to the receiver-operating characteristic (ROC) curve and the Youden index). **(B)** Dasatinib-treated patients were divided into two groups: one with <10% NKG2A+ cells in total CD3^−^CD16^br^CD56^dim^ NK cells (*n* = 11); the other with ≥10% (*n* = 10). *P*-values are calculated using the cumulative incidence approach and the Mentle-Cox method.

Since NKG2A was much down-regulated by dasatinib treatments (Figure 1), we then tested whether lower NKG2A expression in dasatinib-treated patients could be associated with their treatment efficacy (Figure 3). At a cut-off of 10% NKG2A+ cells, all dasatinib-treated patients with fewer than 10% of NKG2A+ cells in all cytotoxic NK cells reached MMR within 12 months after starting on dasatinib. For comparison, the dasatinib-treated patients with 10% or more NKG2A+ NK cells required significantly longer time to reach MMR (Figure 3B). The absolute numbers of NKG2A^+^CD3^−^CD16^br^CD56^dim^ NK cells were also strongly correlated with the length of time to reach MMR. Similarly, at a cut-off of 31 NKG2A+ cytotoxic NK cells per μL, ~90% of the dasatinib-treated patients with <31 NKG2A+ cytotoxic NK cells per μL reached MMR within 12 months, significantly faster than the dasatinib-treated patients with 31 or more NKG2A+ cytotoxic NK cells per μL (Figure 3A). This association was also significant within 18 months of dasatinib treatments.

Of note, this correlation between lower NKG2A+ cell expression and shorter duration to reach MMR in dasatinib-treated patients could not be observed in imatinib-treated or nilotinib-treated patients (data not shown). Furthermore, using linear regression analyses with Pearson's correlation coefficient, we did not find the levels of NKG2A at initial diagnosis to be correlated with the time duration for dasatinib-treated patients to reach MMR (Figure S5). These results revealed that down-regulation of NKG2A by dasatinib helped facilitate anti-cancer responses (Figures 2, 3).

### NK Cytotoxicity Affected by NKG2A Expression

The interaction between NKG2A and HLA-E can block propagation of activating signals in NK cells (18). This interaction plays a crucial role in immune escape (25, 26, 40). Since NKG2A was associated with the effectiveness of dasatinib, we probed the potential role of NKG2A-HLA-E interaction by first examining the levels of HLA-E in CD34^+^ HSCs isolated from healthy adults and from treatment-naive CML patients. The proportions of HLA-E+ HSCs in CML patients were not lower than that in healthy adults (Figure S6). Thus, the association between faster treatment responses and NKG2A down-regulation by dasatinib was mostly likely due to enhanced NK cell cytotoxicity instead.

**Figure 4:**
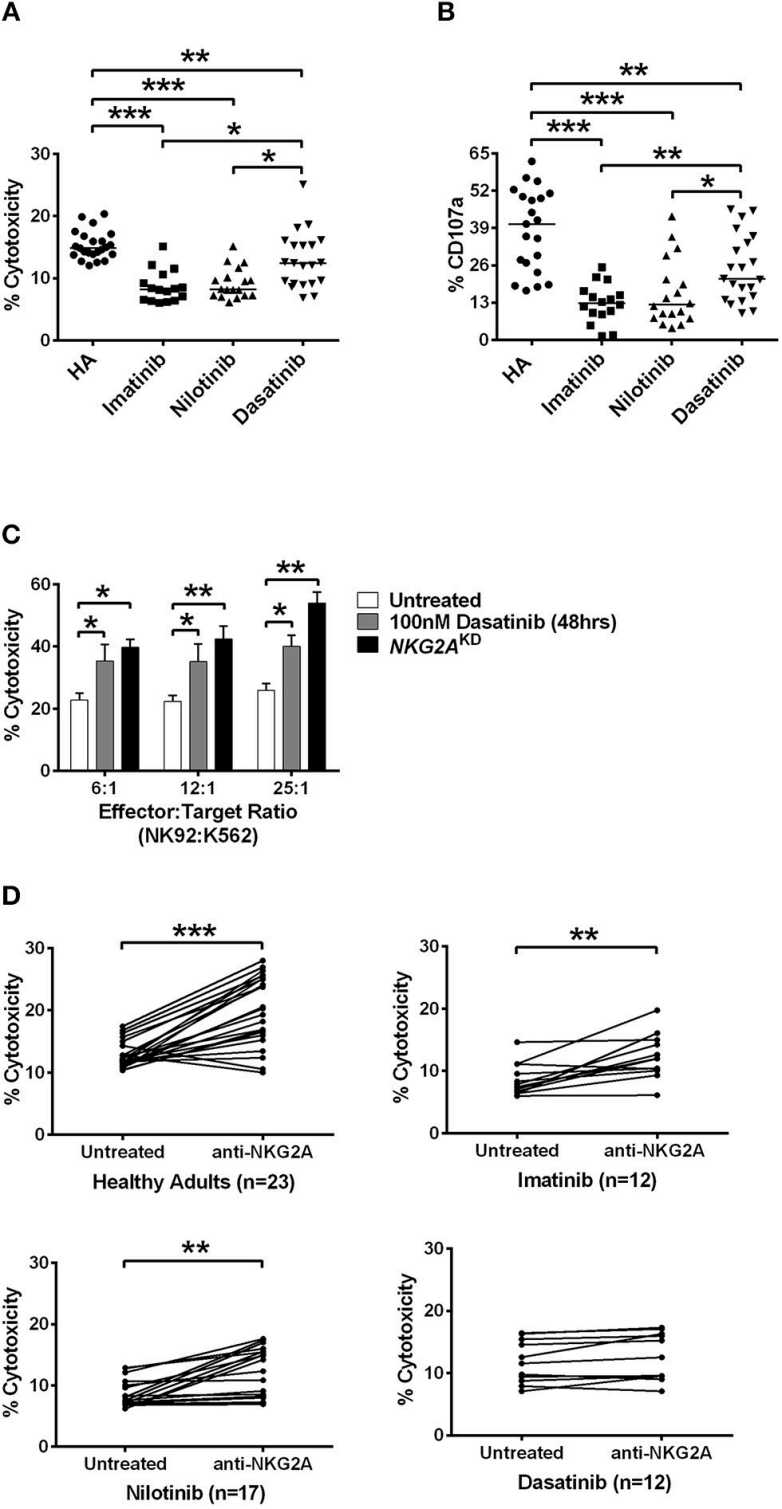
Dasatinib promoted NK cytotoxicity by depletion of NKG2A. **(A)** NK cells were isolated from healthy donors and TKI-treated, CML patients. % cytotoxicity here was assessed by responses of target-effector coculture (an E:T cell ratio of 12:1, with 5 × 10^3^ Target cells/well). The *y*-axis (% cytotoxicity) showed % CFSE+7-AAD+ cells in all CFSE+ cells. Bars denote the median. Significant differences (^*^p < 0.05, and ^***^p < 0.001) were found among NK cells from healthy controls and from patients with different TKI treatments. **(B)** Higher levels of NK degranulation (assessed by %CD107a) in CD3^−^CD16^br^CD56^dim^ NK cells were found in CML patients treated with dasatinib (^*^p < 0.05, ^**^p < 0.01, and ^***^p < 0.001 by non-parametric Mann-Whitney *U*-test). **(C)** At different E:T ratios, % cytotoxicity of the effector NK92 cells was evaluated with dasatinib treatments or with knockdown (KD) of NKG2A. Statistical significance was determined by the unpaired *t*-test. **(D)** NK cells were isolated from healthy subjects and from CML patients treated with imatinib, nilotinib, or dasatinib, followed by 20 min of incubation with anti-NKG2A antibody and then examined by killing assays. Statistical significance was determined by the paired *t*-test (^**^p < 0.01 and ^***^p < 0.001).

Indeed, the NK cells isolated from CML patients on dasatinib generally exhibited significantly higher cytotoxicity against HLA-E-expressing K562 cells (K562-e cells) at an E:T ratio of 12:1, compared with the cytotoxicity of the NK cells isolated from patients on imatinib or on nilotinib. But none of the TKIs could boost NK cytolytic activities to the same levels of the NK cells isolated from healthy donors (Figure 4A). In a different experiment, when we treated NK cells isolated from healthy adults with different TKIs, dasatinib resulted in highest NK cytotoxicity against target K562-e cells *in vitro* (Figure S7).

NK-mediated degranulation was similarly activated by dasatinib. We labeled CD107a in NK cells isolated from healthy adults and from different TKI-treated patients. CD107a is a surrogate marker for NK-cell degranulation. As shown in Figure 4B, the population of CD107a^+^CD3^−^CD16^br^CD56^dim^ NK cells, or degranulating NK cells, was much larger in the dasatinib-treated group (24.9 ± 2.5%), compared to the imatinib-treated (12.7 ± 1.7%; *p* < 0.01) or nilotinib-treated groups (16.3 ± 2.7%; *p* = 0.018). However, the percentages of degranulating NK cells from the dasatinib-treated group were still significantly lower than that from the healthy-control group (Figure 4).

To verify whether NKG2A down-regulation could enhance NK cytolytic function, the expression of NKG2A was abolished by NKG2A-specific shRNA knockdown (KD) in the NK92 cell line. As shown in Figure 4C, the cytotoxicity of effector NK92 cells on target K562-e cells increased significantly after 48-h incubation with dasatinib. Increase in the ratios of effector cell number to target cell number also increased % cytotoxicity, and this was most pronounced in the effector cells with knockdown of NKG2A (Figure 4C). Importantly, NKG2A knockdown in NK92 cells alone elevated NK cytotoxicity substantially.

We further verified whether effects of different TKIs on cytotoxicity was NKG2A-specific. Co-incubation of neutralizing anti-NKG2A antibodies with the NK cells isolated from healthy people significantly enhanced spontaneous NK cytotoxicity against the target cells at an E:T ratio of 12:1 (Figure 4D). When we applied anti-NKG2A blockade to the NK cells isolated from imatinib-treated or nilotinib-treated patients, their NK cytotoxicity also increased. NKG2A blockade restored the cytotoxicity of NK cells isolated from imatinib-treated or nilotinib-treated patients. In contrast, anti-NKG2A antibodies were not able to further enhance the cytolytic activities of the NK cells isolated from dasatinib-treated patients, since the level of NKG2A had been reduced to minimum in dasatinib-treated patients. Taken together, these *in vitro* and *ex vivo* studies demonstrated a critical function of NKG2A in inhibition of NK cytotoxicity (Figure 4).

### P38 MAPK Involved in the Regulation of NKG2A Expression in NK Cells

**Figure 5:**
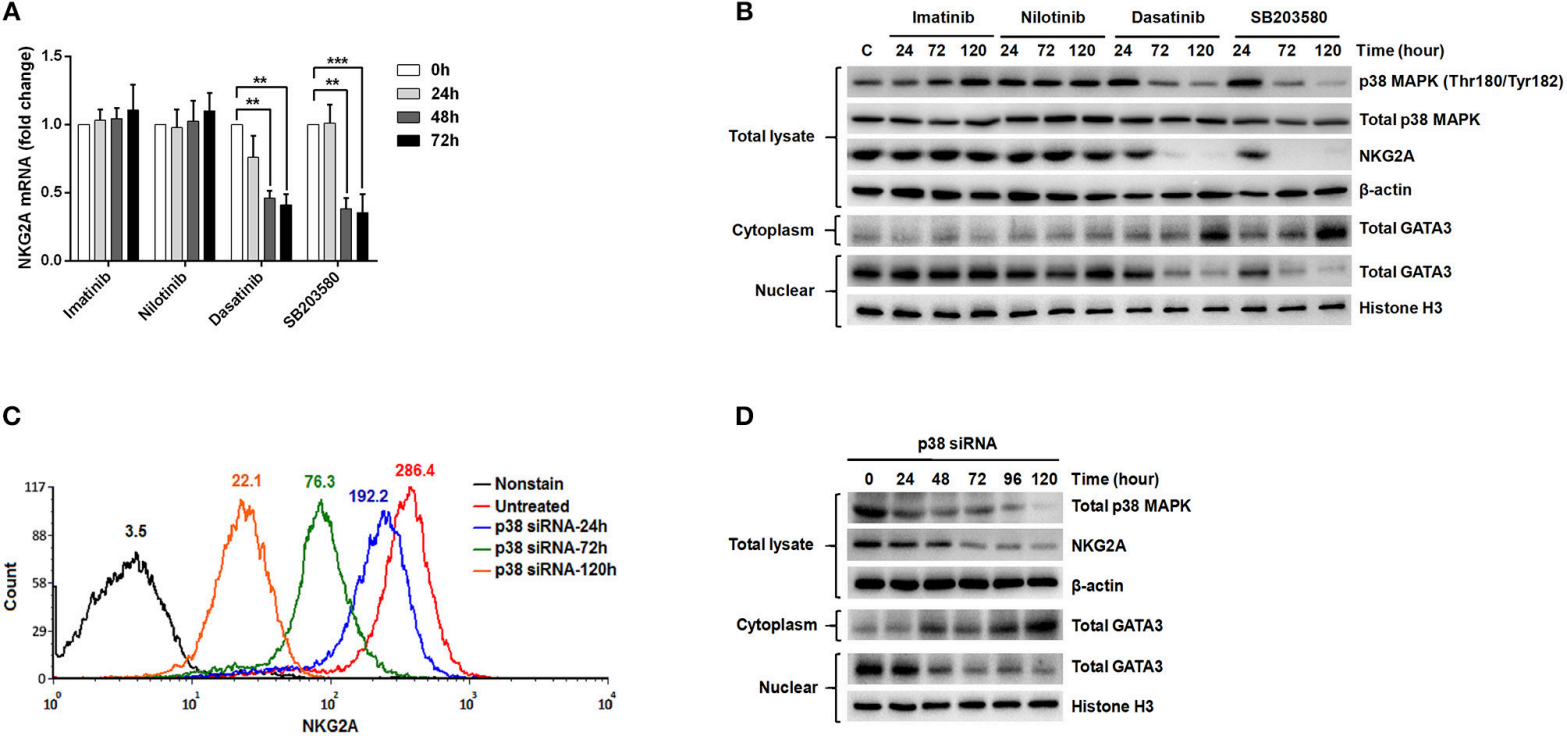
The expression of NKG2A in cytotoxic NK cells was affected by p38 MAPK activities and GATA-3-mediated transcription. **(A)** NK92 cells were treated with imatinib (2 μM), nilotinib (2 μM), dasatinib (100 nM), and SB203580 (1 μM) over time, and NKG2A transcripts were quantified by RQ-PCR. **(B)** NK92 cells were harvested after treatments with TKIs and SB203580 at indicated time-points. Western blot analysis was performed on cell lysates (10^6^ cells/samples) using anti-phosphorylated p38 (Thr180/Tyr182) and anti-p38 Abs (top panels) sequentially on the same membrane, anti-NKG2A (middle panel), and anti-actin (an internal control) (bottom panel) sequentially on duplicate membranes. For GATA-3 protein analysis, cytosol and nuclear lysates were extracted individually for immunoblotting using anti-GATA-3. Anti-Histone H3 was used to quantify nuclear proteins. **(C,D)** P38 knockdown was performed in NK92 cells over time, followed by MFI determination for NKG2A by flow cytometry, and by Western blot analyses for p38, NKG2A, and GATA-3. The numbers on a representative histogram indicate the MFI of p38 protein. Statistical significance was determined by the paired *t*-test (^**^p < 0.01 and ^***^p < 0.001).

According to the previous studies (32, 34), GATA-3 is an important transcription factor that regulates *NKG2A* gene expression. Translocation of GATA-3 into the cell nuclei is dependent on its serine phosphorylation by p38 kinase. To probe into how NKG2A expression could be regulated in NK cells, we examined the effects of ABL TKIs on p38, GATA-3, and NKG2A transcription. We challenged NK92 cells with TKIs and SB203580, a specific inhibitor of p38 kinase, and then examined how NKG2A transcript levels could be affected using RQ-PCR. As shown in Figure 5A, dasatinib or SB203580 treatments for 48 and 72 h suppressed the expression of NKG2A to one-third or even one-quarter, compared to imatinib or nilotinib treatments that exerted absolute no effects on NKG2A transcripts over time.

In a parallel experiment, Western blot analysis of the lysates of NK92 cells showed strikingly diminished NKG2A protein after incubation with dasatinib or SB203580. The decrease in NKG2A protein expression was parallel to the drop in phosphorylated p38 MAPK expression (Figure 5B). As phosphorylated p38 mediates phosphorylation of GATA-3 at serine residue 308 (p-GATA-3) and affects nuclear import of GATA-3 (32), we indeed observed drastically decreased GATA-3 expression in the cell nuclei and increased GATA3 expression in the cytoplasm after treatments with dasatinib or SB203580. Importantly, the expressions of phosphorylated p38 and NKG2A, as well as subcellular localization of GATA3, were not affected by imatinib or nilotinib (Figure 5B).

To validate the effect of p38 on NKG2A expression, we knocked down p38 in NK92 cells, and then measured NKG2A by immunocytometry (Figure 5C) and immunoblotting (Figure 5D). Knock-down of p38 reduced surface expression of endogenous NKG2A over time (Figure 5C). Moreover, the decrease of NKG2A expression was preceded by a decrease of GATA-3 in the cell nuclei (Figure 5D). The level of cytoplasmic GATA-3 was elevated, when p38 expression was knocked down (Figure 5D). Taken together, NKG2A down-regulation by dasatinib resulted from dasatinib-specific blockade of p38 activities and subsequent GATA-3 import to the cell nuclei (Figure 5). Thus, the process by which dasatinib shaped NK profiles in CML is dynamic, and involved regulation at the transcriptional level.

## Discussion

This study uncovered an immunomodulatory mechanism of dasatinib through dasatinib-specific suppression of inhibitory NK receptor NKG2A. Like the other TKIs imatinib and nilotinib, dasatinib is an effective inhibitor for BCR-ABL formation. This additional function of dasatinib revealed in this study explains why successful anti-CML treatments by dasatinib often accompany peripheral NK lymphocytosis (Figures 1A, 2) (11–13).

This work started by surveying expressions of 7 major activating and inhibitory NK receptors (NKG2D, NKp30, DNAM-1, 2B4, NKG2A, KIR2DL1, and KIR3DL1) in CML patients during their initial diagnosis and at different treatment stages. Similar to others' findings, we also found that newly-diagnosed CML patients expressed fewer activating receptors (i.e., NKG2D, NKp30, and DNAM-1 in Figure 1A) on their NK cell surface. At initial diagnosis, there were no apparent differences in the proportion of cytotoxic NK cells in total lymphocytes compared to healthy controls. However, the expressions of inhibitory NK receptors (i.e., KIR2DL1 and KIR3DL1 in Figure 1A) were not significant except the protein expression of NKG2A, when compared to healthy controls (Figure 1A). When comparing the impacts of different TKI treatments on these NK receptors, dasatinib uniquely down-regulated NKG2A (Figures 1B,C). Further dissection on the possible mechanism showed that dasatinib inhibited p38 MAPK signaling, which consequently affected nuclear import of GATA-3 and transcriptional activities of GATA3 for NKG2A (Figure 5). This allowed dasatinib to promote NK cytotoxicity and degranulating ability by selective suppression of NKG2A, as demonstrated *in vitro* and *ex vivo* (Figure 4). These basic research findings provided an explanation as to why dasatinib-treated CML patients reached effective treatment responses faster in our cohort (Figures 2-4).

NK cells utilize a diverse array of inhibitory and activating receptors for recognition of target cells and for control of NK activation and cytotoxicity. The surveyed activating receptors on the NK cell surface (NKG2D, NKp30, DNAM-1, and 2B4) are up-regulated by ligand binding and signals from cellular stress, and could trigger positive signals for killing. In contrast, the surveyed inhibitory receptors on the NK cells (NKG2A, KIR2DL1, and KIR3DL1) are specific for different HLA–class I molecules, and could induce negative signaling for killing upon binding to HLA-I. Integration of these opposite signals determines the status of NK cell activation (41).

Though defects of NK cells in cell number and/or functions have been noted upon initial diagnosis of CML-CP (42, 43), successful therapeutic interventions could revert or even restore expressions of NK receptors to the levels close to that in healthy people (17). For example, reversion of the expressions of NK receptors to normal levels is observed in breast cancer patients who had not relapsed after tumor removal over 5 years ago (44). Similarly, in our study, three of the four surveyed activating receptors (NKG2D, NKp30, and DNAM-1) rebounded in surface expression after successful TKI treatments for CML (Figure S4), and the number of cytotoxic NK cells expressing these activating receptors also reverted or increased simultaneously. Such reversion of effector molecules on the NK cell surface by TKIs reflects a status of disease control.

Notably, dasatinib triggered exacerbated reversion of NKG2A expression to very low levels in the treated CML patients (Figure 1); this was correlated with faster treatment responses by dasatinib (Figure 3). The anti-cancer mechanisms that dasatinib utilized through NKG2A are immune-mediated, with the two lines of evidence. First, in our CML study, suppression of NKG2A by dasatinib enhanced NK cytotoxicity (Figure 4). A pre-clinical study for chronic lymphocytic leukemia similarly finds that NKG2A is suppressed by Monalizumab (a mAb against NKG2A), resulting in overall enhancement of anti-leukemia activities (45). Second, though binding of HLA-E to NKG2A can induce inhibitory signals that suppress cytokine secretion and cytotoxicity of the effector cells against cancerous cells, inhibition of NKG2A by dasatinib could block this route of immune escape. Thus, different immunomodulatory mechanisms through NKG2A may be triggered by anti-cancer therapeutics like dasatinib.

Nonetheless, how dasatinib affects cancer immunity remains unclear. In CML, we found that dasatinib-specific inhibition of p38 and GATA-3 transcriptional activities depleted NKG2A, leading to higher NK cytotoxicity and stronger anti-CML responses. Some reports also show that a short exposure to dasatinib can activate NK cells *in vitro* (14, 46) and in a mouse model *in vivo* (47). Clinically, a majority of CML or Ph+ ALL patients with long-term administration of dasatinib show sustained control of leukemia and molecular responses (11, 12, 48). But there are also reports suggesting that dasatinib is immunosuppressive on NK cells *in vitro* and in mouse models, and that dasatinib-mediated inhibition of a broad spectrum of protein kinases (including Src family kinases (SFKs), Tec family kinases, MAPK family, and BCR-ABL kinase) might be involved (22, 49–51). These contradicting observations point to an intricate balance between activating and inhibitory NK receptor signaling, especially in distinct populations of target cells (52). Future work on quantification of the balance between activating and inhibitory NK signaling may help clarify these contradicting issues (53).

## Ethics Statement

The study was approved by the Mackay Memorial Hospital institutional review board (18MMHIS113) and was carried out in accordance with the principles of the Declaration of Helsinki. Genetic transduction procedures were performed according to Taiwan Ministry of Science and Technology legislation. Permission for the projects was granted by the MacKay Memorial Hospital.

## Author Contributions

M-CC and CC developed the study, performed experiments, analyzed data, and wrote the paper. KH contributed to the writing of the paper. C-WK performed experiments. H-IC and Y-NH performed bioinfomatic analyses. M-CC, Y-FC, and K-HL contributed to patient care.

### Conflict of Interest Statement

The authors declare that the research was conducted in the absence of any commercial or financial relationships that could be construed as a potential conflict of interest.
