# Supplementary material for: NKG2A Down-Regulation by Dasatinib Enhances Natural Killer Cytotoxicity and Accelerates Effective Treatment Responses in Patients With Chronic Myeloid Leukemia
Source: Front Immunol. 2019 Jan 17;9:3152. doi: 10.3389/fimmu.2018.03152 (PMC6344416; doi:10.3389/fimmu.2018.03152)
Supplement: Supplementary file 1 [file Data_Sheet_1.docx]

**NKG2A Down-regulation by Dasatinib Enhances Natural Killer Cytotoxicity and Accelerates Effective Treatment Responses in Patients with Chronic Myeloid Leukemia**

Ming-Chin Chang^1,2^．Hung-I Cheng^3^．Kate Hsu^4^．Yen-Ning Hsu^3^．Chen-Wei Kao^5^． Yi-Fang Chang^1,2,5^．Ken-Hong Lim^1,2,5^．Caleb Gonshen Chen^1,2,5^

**Supplementary Figures**

**Figure S1**

**
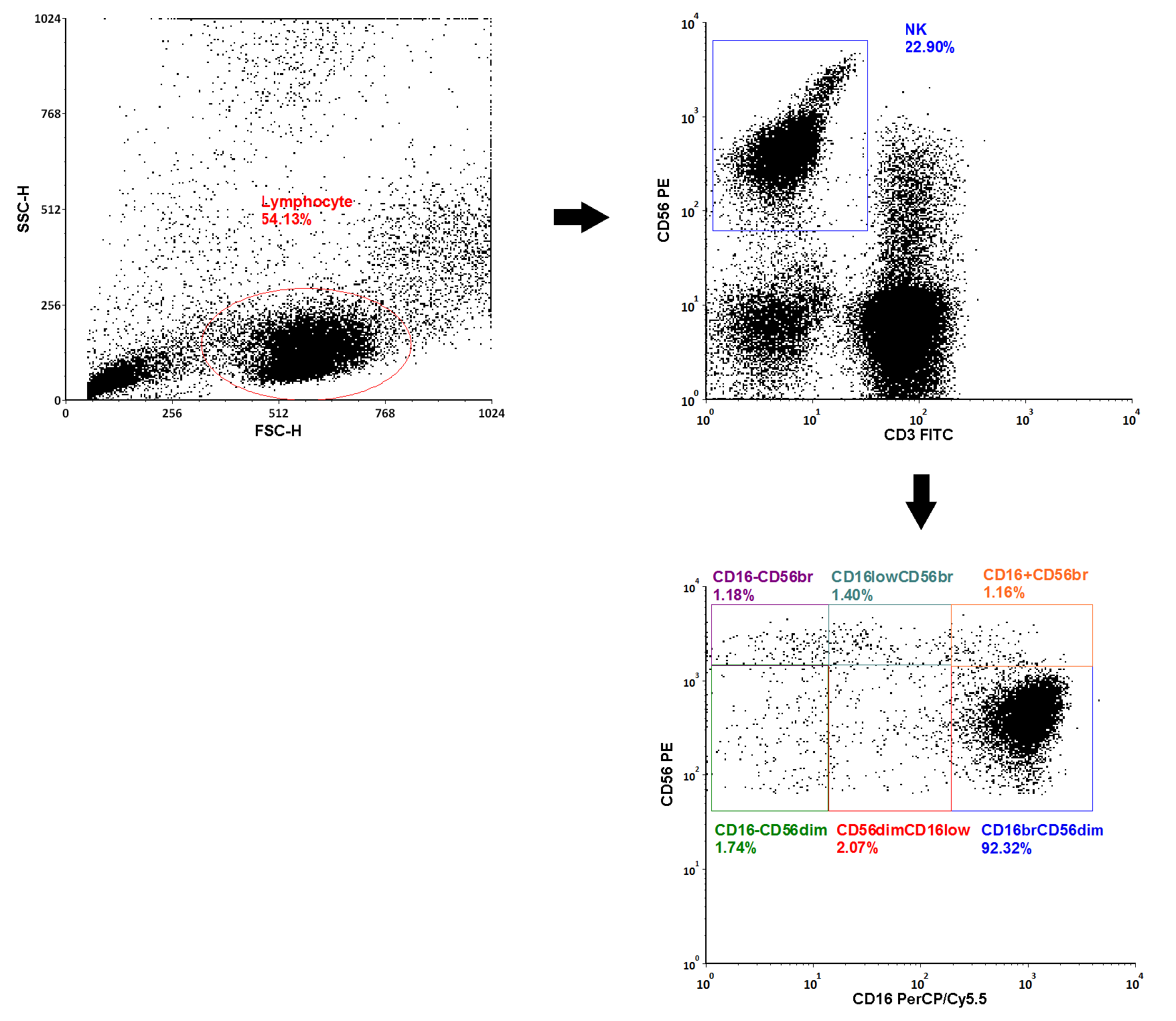
**

**Figure S1.** Our Gating strategy for identifying CD3^-^CD16^br^CD56^dim^cell population. Total lymphocytes were first divided into CD3^-^ and CD3+ populations. CD16 and CD56 staining allowed gating for CD16^br^CD56^dim^ cells in the CD3^-^ cell populations.

**Figure S2**


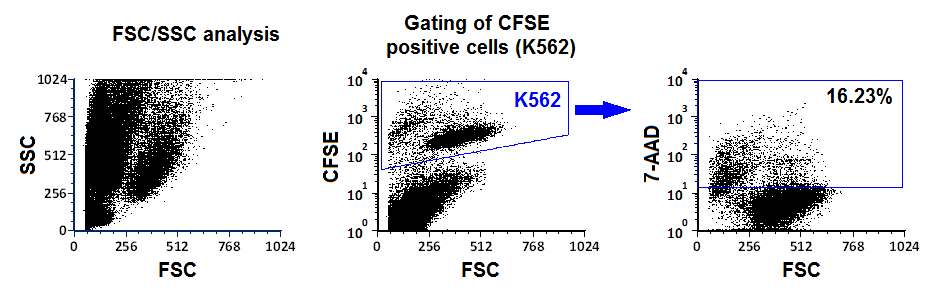


**Figure S2.** Cytotoxicity assayed by CFSE and 7-AAD staining. CFSE-stained K562 cells were co-cultured with NK cells at an E:T ratio of 12:1. The CFSE-stained K562 cells were first gated by FSC and SSC, where 20,000 events were captured. The numbers inside the gate denoted the percentage of 7-AAD positive cells.

**Figure S3**





**Figure S3.** Comparison of the absolute numbers of CD3^-^CD16^br^CD56^dim^ NK cells/μL in healthy adults and in CML patients at initial diagnosis and on different TKI treatments. Statistical significance was determined by the *t*-test or nonparametric Mann-Whitney U-test. **p<0.01, ^#^p<0.001.

**Figure S4**





**Figure S4.** The three TKIs exerted differential effects on expressions of activating receptors (NKG2D, NKp30, DNAM-1, and 2B4). Statistical significance was determined by the Student’s *t*-test. *p< 0.05, **p<0.01. The Mean fluorescent intensities (MFI) reflect relative protein levels.

**Figure S5**





**Figure S5.** By linear regression analysis with Pearson’s correlation coefficients, no significant correlation was found between NKG2A expression levels at the time of initial diagnosis (treatment-naïve) and at the time when patients reached MMR.

**Figure S6**





**Figure S6**. The levels of HLA-E in CD34+CD45+ hematopoietic stem cells from the bone marrow of healthy donors and the CML patients at their initial diagnosis were not different. The surface levels of HLA-E were determined by flow cytometry, and shown as MFI.

**Figure S7**





**Figure S7.** The three BCR-ABL inhibitors exerted differential effects on NK-cell cytotoxicity. NK cells isolated from healthy donors were pre-incubated with imatinib, nilotinib, or dasatinib for 48h, and then co-incubated with target cells (5 × 10^3^ cells/well) at an E:T ratio of 12:1. The *y*-axis shows the percentage of CFSE^+^7-AAD^+^ cells in all CFSE^+^ cells. Bars denote the median. Statistically significant values (***p < 0.001) was determined by the non-parametric Mann-Whitney U-test.
